# Direct breastfeeding frequency of late preterm and term infants in the neonatal intensive care unit and availability of mother’s own milk at six months of age. a retrospective cohort study

**DOI:** 10.1038/s41372-024-01972-w

**Published:** 2024-04-27

**Authors:** Amanda E Smith, Erin Sweigart, Kimberly Falatic, Dena Stuart, Heidi Szugye, Suet Kam Lam, Hany Aly, Anirudha Das

**Affiliations:** 1 Department of Neonatology, Cleveland Clinic Children’s Hospital, Cleveland, OH USA; 2 Department of Childbirth Education, Cleveland Clinic, Cleveland, OH USA; 3 Breastfeeding Medicine, Department of Primary Care Pediatrics, Cleveland Clinic Children’s, Cleveland, OH USA

**Keywords:** Paediatrics, Risk factors

## Abstract

**Background:**

Mother’s Own Milk (MOM) reduces the risk of complications in premature infants. Breastfeeding rates for late preterm and term infants in the neonatal intensive care unit (NICU) are significantly lower than that of breastfed healthy term newborns at 6 months of age.

**Design:**

This was a retrospective cohort study of neonates born at 34 weeks 0 days or later. Infants who were directly breastfed in the NICU and were discharged on breast milk were included. Logistic regression modeling was used to determine the significance of association.

**Results:**

171 mother-infant dyads were included. After adjusting for confounders, the number of breastfeeding attempts during the NICU stay was significantly associated with the availability of MOM at six months of age (*p* = 0.003, 95% CI 1.02 to 1.14).

**Conclusion:**

This study is the first to show an association between the number of direct breastfeeding attempts in the NICU and availability of MOM at six months of age.

## Introduction

Mother’s own milk (MOM) is widely regarded as the best nutrition for infants as it decreases feeding intolerance, late onset sepsis, sudden infant death syndrome, and mortality, among other benefits [1–5]. Premature neonates who receive MOM have decreased risks of complications such as necrotizing enterocolitis, neurocognitive delays, retinopathy of prematurity, and chronic lung disease [1–4].

The American Academy of Pediatrics and the World Health Organization recommend exclusive breastfeeding until 6 months of age for all newborns, and continued breastfeeding for 2 years of age and beyond [6, 7]. Even partial breastfeeding has been shown to be beneficial to children in terms of reducing infections, eczema, and asthma [8, 9]. Breast milk provides the best nutrition, immunity, metabolism, growth, and development for infants [10]. Breast milk contains hormones, neuropeptides and growth factors that are beneficial to growth, development, and self-regulation of food intake, providing the breastfed infants with significant advantages compared to their formula-fed counterparts [11].

Breastfeeding rates for infants admitted to the Neonatal Intensive Care Unit (NICU) are significantly lower than that of healthy newborns with exclusive breastfeeding rates reported of only 19.4% at 6 months age [12, 13]. There are several factors that are associated with this lower rate of exclusive breastfeeding such as immature feeding pattern of prematurity, delayed initiation of direct breastfeeding (DBF), higher stress levels in the mother, lack of bonding, lower rates of skin-to-skin care, and prolonged hospitalization [12, 14].

There is a lack of research studies examining maternal, infant, and health system factors associated with continued rates of availability of MOM in late preterm and term infants discharged home from the NICU. Previous studies have documented that for late preterm infants, the focus is on growth of the infant and therefore DBF is not prioritized over supplementing calories [15]. It is not clear whether encouraging, supporting, and trying to establish breastfeeding in the late preterm and term infants prior to discharge from the NICU has long term benefits for the infant in terms of receiving MOM. The registered nurses caring for babies in the Cleveland Clinic NICU receive 5 hours of shadowing and training from inpatient lactation consultants in the NICU and on the mother-baby unit. This education includes completing a checklist of basic breastfeeding skills upon hire. Additional lactation education for nurses is optional. Lactation consultants (LC) provide in-person support to mothers during the week and are on-call over the weekend. Mothers who desire to do DBF are automatically offered at least one lactation consultation visit. Subsequent visits can be initiated by the mother, the LC or the nurse based on the need.

The objective of this study was to evaluate the relationship between the frequency of direct breastfeeding of late preterm or term infants while in the NICU and the availability of any amount of MOM for the infant at 6 months of age after adjusting for the known confounding variables. A duration of six months was chosen as it is the minimum recommended length of time to receive breast milk.

## Methods

### Study design

This was an observational retrospective cohort study approved by the Institutional Review Board (IRB) of the Cleveland Clinic Foundation. The need for informed consent was waived by the IRB.

### Setting

The study was conducted in two level three Neonatal Intensive Care Units in an urban setting on admitted neonates who met the inclusion criteria between January 2020 and April 2021. Data was collected retrospectively by chart review.

### Participants

Infants born within the Cleveland Clinic Hospital System requiring admission to the Neonatal Intensive Care Unit (NICU) and fulfilling the following study criteria were included.

#### Inclusion criteria

- Infants born at 34 weeks 0 days or later discharged from the NICU and had followed up within the same Health System.
- Mother initiated breastfeeding in the NICU.
- Infants receiving human milk at discharge.

#### Exclusion criteria

- Infant with NICU stay for less than 5 days.
- Medical contraindication to breastfeeding.
- Mother not providing breast milk at discharge.
- >1 readmission or >2 Emergency Department visits by the infant in the first 6 months of life.
- Multiple live births.
- Dyads without information about breast milk feeding at 6 months.

### Variables

This was a retrospective cohort study where infants admitted to the NICU who met the study criteria, were retrospectively followed-up after discharge to determine whether they were receiving any amount of MOM (either direct breastfeeding or bottle feeding) as documented at the 6 month visit by their primary care physician.

### Data source/measurements

Data were obtained from retrospective review of infant charts included in the study. Measurements of the variables were standardized and self-explanatory. Mother’s age was considered at the time of delivery. Gravida included the current pregnancy while parity did not include the delivery of the infant admitted to the NICU. The days on respiratory support included the number of days infant was on nasal cannula, Continuous Positive Airway Pressure (CPAP), Ventilator and High Frequency Oscillation. The vaginal delivery type included forceps and vacuum deliveries as well. The number of lactation consultation (LC) encounters were based on their notes written on the infant’s chart.

### Study size

To demonstrate a difference of 20% between the groups in the outcome variable, for a medium size effect, we needed a sample size of 124 dyads. We considered 171 mother-infant dyads based on the uncertainty of number of variables that may qualify for modeling and for a moderate size effect.

### Statistical methods

SPSS software was used for data analysis (IBM SPSS version 27, 2020). We used convenience sampling method. Median and interquartile range were reported to describe the demographic as well as outcome variables. Missing data were not analyzed. Median and interquartile range were reported for the non-normal data with categorical variables reported as percentages. For univariate analysis, logistic regression was used for categorical variables and simple linear regression for continuous variables. All variables with a significant association (*p* value of <0.05) with the outcome were considered confounders and analyzed using multivariable regression model to determine the significance of association. The model was examined for goodness of fit and data reported as odds ratio and confidence interval.

## Results

A total of 184 infant-mother dyads were identified, of which six multiples (12) were excluded and one dyad did not qualify because of the lack of available information regarding breast milk feeding at 6 months. Among the 171 (184–13) infants included in the final analysis, the median gestational age at birth was 35 weeks with a birth weight of 2370 grams (Table 1). At two months the number of infants getting MOM was 96/171 (56.1%) which decreased to 69/170 (40.5%) at 4 months and 57/171 (33.3%) at 6 months of age.

**Table 1:** Unadjusted demographic and outcome characteristics the groups.

Characteristic	Receiving breast milk at 6 months (*n* = 57) (Median and Interquartile range)	Not receiving breast milk at 6 months (*n* = 114) (Median and Interquartile range)	*P* value (Odds ratio, Confidence interval)
Birth weight in grams	2452 (2140–3085)	2332 (1980–2760)	0.07
Gestational Age at birth (in weeks)	35.1 (34.4–37.5)	35.1 (34.3–37.2)	0.43
Caucasian race	43.5%	39.5%	0.85
Apgar 1 minute	8 (7-8)	8 (6–8)	0.06
Apgar 5 min	9 (8-9)	8 (8-9)	0.01* (1.7, 1.1–2.6)
Delivery type (Vaginal)	51%	33%	0.02* (2.1, 1.09–4)
Male gender	68.4%	57.9%	0.24
Mother’s age in years	33 (29–34)	30 (25–34.2)	0.02* (1.07, 1.01–1.14)
Length of stay in days	10 (7–13)	10 (7–13.2)	0.63
Smoking in pregnancy	1.8%	4.4%	0.2
Gravida	2 (1–3)	2 (1–3)	0.42
Parity (prior to delivery)	1 (0–1)	1 (0–1)	0.65
Days spent on respiratory support	1 (0–2)	0 (0–1)	0.07
Number of lactation consultant visits during the NICU stay	5 (4–7)	4 (3–6)	0.02* (1.17, 1.02–1.3)
Number of breastfeeding attempts during the NICU stay (Total)	7 (3–16.5)	2 (1–6.2)	<0.001* (1.12, 1.06–1.1)

*P value < 0.05.

Overall, the median length of stay (LOS) was 10 days, maternal age was 31 years and infants were on respiratory support for less than a day. The median number of LC visits were 4.5 (Range 0–15).

The infants receiving MOM at 6 months had a higher birth weight and gestational age, but the difference was not significant. The Apgar scores at 1 and 5 min were lower in the group that received any MOM at six months but only the five-minute Apgar was significantly higher in the group that did not receive MOM at 6 months. The mothers of the infants receiving MOM were significantly older than those not receiving MOM (33 vs 30 years, *p* < 0.03). Significantly higher number of infants who received MOM at 6 months were born via vaginal delivery (51% vs 33%). Infants in the group who continued to receive MOM at 6 months had more males (68.4% vs 57.9%), mothers with lower rate of smoking during pregnancy (1.8% vs 4.4%) but these differences were not significant. There was no significant difference between the groups in terms of LOS in the NICU, gravida/parity of mother and infant’s number of days spent on respiratory support. The number of LC visits were significantly higher in the group that received MOM at 6 months (5 vs 4, *p* = 0.02, OR 1.17, CI = 1.02–1.3).

Using logistic regression modeling, we adjusted for the variables that were significantly associated with infants receiving MOM at 6 months (*p* < 0.05): Apgar at 5 min, vaginal delivery, mother’s age, and lactation visits during the NICU stay.

**Fig. 1:**
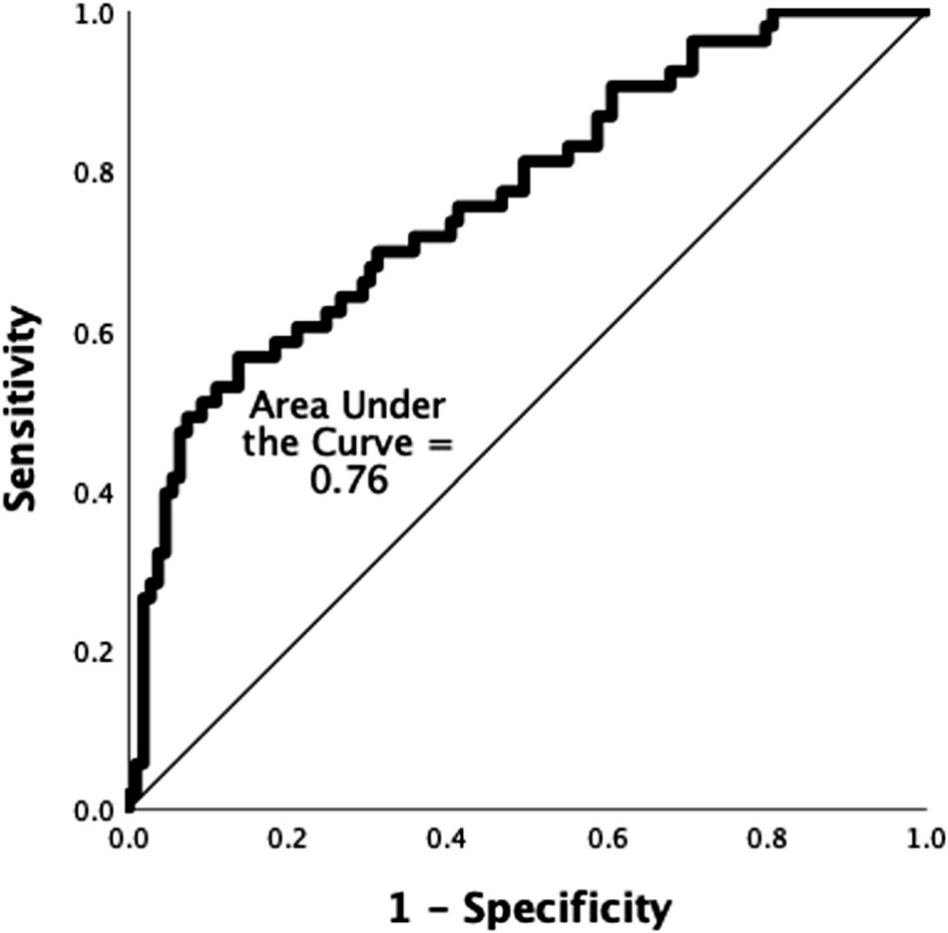
The ability of number of attempts at breastfeeding during the NICU stay to predict the outcome, availability of mother’s own milk at 6 months age.

The model had appropriate goodness of fit as the Omnibus test of model coefficient showed a high Chi squared value of 33.84 which was also significant (*p* < 0.001) and the Hosmer and Lemeshow test showed a *p* value of 0.5. On logistic regression analysis, when adjusted for the variables, the number of breastfeeding attempts during the NICU stay were significantly associated with the infant availability of MOM to the infant at six months of age (*p* = 0.003, 95% CI (Confidence Interval,) 1.02 to 1.14) (Table 2). A receiver operating characteristic curve (ROC) plotted with the availability of MOM at six months of life as the binary outcome variable showed the very good discriminatory ability of the number of breastfeeding attempts during the inpatient stay of the infant (Area under the curve of 0.76) (Fig. 1). The other variables Apgar score at 5 min, vaginal delivery, age of the mother and lactation encounters did not have significant association with the outcome variable in the model. There was 9.5% increase in odds of the infant getting MOM at 6 months if the infant is directly breastfed in the NICU when adjusted for the confounding variables.

**Table 2:** Multiple logistic regression analysis of the independent variables demonstrating their relationship with the outcome variable, rate of availability of MOM adjusted for the effect of the covariates.

Characteristic	Beta (log odds)	df	Significance	Exp(B)	CI upper	CI Lower
Age of mother	0.065	1	0.069	1.067	0.995	1.145
Vaginal delivery	0.504	1	0.204	1.655	0.760	3.606
Total APGAR Score at 5 min	0.457	1	0.054	1.579	0.992	2.514
Number of times breastfed in the NICU	0.083	1	0.003*	1.086	1.028	1.148
Number of lactation consultant visits	0.118	1	0.152	1.125	0.957	1.322

*df* degree of freedom, *CI* confidence interval, *Exp(B)* exponent for Beta value (Odd’s ratio).

*P value < 0.05.

## Discussion

This study demonstrated that number of direct breastfeeding attempts in the NICU is predictive of the availability of MOM at 6 months corrected age after adjusting for age of the mother, type of delivery, APGAR score at 5 min and number of inpatient LC encounters. There was a 9.5% increase in odds of the infant getting MOM at 6 months for every breastfeeding attempt in the NICU.

Direct breastfeeding can be challenging in late preterm infants who may not be very alert necessitating pumping of human milk to protect milk production and provide supplemental feeds to the infant. The providers may also not be comfortable in directly breastfeeding a sick term infant who is recovering, to ascertain that the infant meets the fluid goals. To the best of our knowledge, this is the first study to show that increased DBF in late preterm and term infants during the NICU stay is associated with prolonged availability of MOM for the infant highlighting the benefit of direct breastfeeding early on. Since late preterm and term infants are usually not tested for development at 18-24 months, it is not possible to predict an association of DBF in this population with long term neurodevelopmental outcome.

In our study, vaginal birth was associated with a significantly higher chance of receiving MOM at 6 months age although the association was not significant when adjusted for other variables. This is consistent with a study conducted by Jiang (2022) who demonstrated a 56% higher chance of baby receiving MOM at 6 months if the infant was born by normal vaginal delivery [12]. The study also showed an association between APGAR score and breastfeeding at 6 months which is similar to our study [12].

In a study by Perrella (2022), based in Western Australia, 59% of infants continue to receive MOM at 3 months corrected age after discharge from the NICU [16]. Hannan (2018), showed that 64% of late preterm infants were still receiving MOM (either directly or via bottle) at 10 weeks corrected gestational age [15]. Finally, in a United States based study, Briere (2016) showed 51%, 26% and 23% of the infants received breast milk at 1, 4 and 8 months corrected gestational age, respectively [17]. The percentage of infants receiving MOM feeding at 2, 4 and 6 months in our study was similar to the above-described studies (56%, 40% and 33% respectively).

Previous research has shown that younger maternal age is a risk factor for decreased rates of continuation of breastfeeding till 6 months of age [1, 15, 18, 19]. The findings of our study were consistent with the previous studies as it showed that age of the mother was significantly less in infants not receiving MOM at 6 months although the difference did not persist after adjusting for confounding variables. Our study included mothers who initiated DBF in the NICU and their infant received human milk at discharge. This could have resulted in the exclusion of younger mothers who were not interested in breastfeeding being excluded from our study as evidenced by an average age of 30 years all study participants and no participants less than 25 years of age. Casey (2018) has already shown that older mothers (mean age 31 years) had a higher chance of directly breastfeeding their preterm infants (<34 weeks gestational age) at discharge from the NICU [4]. The age profile of mothers of our study was similar to the studies by Gianna (2019), which included mothers aged 35.5 years (± 4.6), Hannan (2017) where majority of the mothers were in the range of 25–34 years and Jonsdottir (2020) with average maternal age of 30 years [1, 15, 19].

Briere (2016) found a positive correlation between direct breastfeeding in the NICU for infants < 32 weeks and/or <1500 g and breastfeeding rates at 1 month and at 4 months corrected gestational age, post-discharge [17]. These results, combined with the results of our study, allow us to infer that regardless of gestational age, babies who are directly breastfed in the NICU are likely to receive MOM longer than babies exclusively receiving expressed breast milk while in the NICU. These results also align with breastfeeding studies in general infant population that have found that mothers who directly breastfeed their infant provide MOM for a longer duration than mothers who exclusively pump and feed expressed breast milk [3, 20].

Casey (2018) also reported that premature infants who directly breastfed for their first oral feed are more likely to receive MOM at discharge from the NICU [4]. Our study considered the number of breastfeeding attempts as opposed to the first oral feed. This suggest that not only starting DBF early but also encouraging mothers to directly breastfeed more frequently has sustained effects on the infant for up to six months corrected age in terms of access to MOM.

Furthermore, it is important to consider the potential impact of access to LC visits and the education and support from the NICU nurses. Mercado (2019) compared two NICUs with or without LC support and demonstrated that the unit with LC support had an increased number of direct breastfeeding attempts [21]. The findings were consistent with this study. There was a weak correlation (0.16) between the number of LC visits and the number of DBF attempts made in the NICU although it was significant (p = 0.03). The number of lactation visits was associated with increased number of direct breastfeeding attempts in the NICU and availability of MOM at 6 months, but this association was not significant when adjusted for other variables. This demonstrates the complex nature of this association wherein it is difficult to determine the reason behind the increased LC visits in the NICU. Although a greater number of LC visits in the NICU results in higher DBF rate, the increased number could be due to the interested mothers who are struggling to breastfeed requiring more support who eventually cannot sustain the production of their own milk due to factors such as psychological distress or lack of family support, which were not considered in this study.

Mothers should be encouraged to do DBF while in the NICU as there are many health benefits for their baby. The number of DBF attempts in the NICU is associated with a higher rate of providing any breast milk by the mother at six months of age. Additional research is needed to identify interventions that promote and provide support for mothers with infants in the NICU interested in breastfeeding without causing psychological harm to mothers that are not able or interested in breastfeeding. Potential strategies to explore include increasing education for NICU staff and parents about the benefits and feasibility of breastfeeding an infant in the NICU; increased lactation support; and sufficient nursing staffing to support early initiation of DBF. Finally, public and employer policy changes should be explored that would allow mothers access to paid time off while their infant is in the NICU.

## Limitations

Since the amount of breast milk available to the infant at 6 months was not quantified in this study it is not possible to deduct a statistical long-term affect. Although breastfeeding is beneficial beyond the first 6 months of age; this study did not consider breast milk feeding rates at one to two years of age. Retrospective studies have inherent bias which is difficult to determine and eliminate. While skin-to-skin care is recommended and encouraged to support direct breastfeeding, this study did not examine this data. Breastfeeding support outside of the Cleveland Clinic system, such as through lactation consultants, pediatrician clinics, or support groups were not examined during this study. Covid-19 may have affected breastfeeding in the NICU during part of the study period as there were restrictions on hospital visitation during the peak incidence, but it is beyond the scope of this study to determine the effect of that on individual subjects.

## Conclusions

This study demonstrates the importance of DBF in the NICU for late preterm and term infants. Increased number of DBF attempts in the NICU is associated with higher rate of any amount of MOM feeding at 6 months age of the infant. The study showed that the need for supplementation and bottle feeding of late preterm infants should be finely balanced with encouragement for DBF. Prospective studies are needed to confirm the findings of this study and identify strategies to support mothers who face challenges in breastfeeding while their infant is in the NICU.

## Data Availability

The data that support the findings of this study are available from the corresponding author upon reasonable request.
